# 3-[2-(4-Fluoro­phen­yl)-2-oxoeth­yl]-5,5-di­phenyl­imidazolidine-2,4-dione

**DOI:** 10.1107/S1600536814001743

**Published:** 2014-01-31

**Authors:** Joel T. Mague, Alaa A.-M. Abdel-Aziz, Adel S. El-Azab

**Affiliations:** a Department of Chemistry, Tulane University, New Orleans, LA 70118, USA; b Department of Pharmaceutical Chemistry, College of Pharmacy, King Saud University, Riyadh 11451, Saudi Arabia; c Department of Medicinal Chemistry, Faculty of Pharmacy, University of Mansoura, Mansoura 35516, Egypt; d Department of Organic Chemistry, Faculty of Pharmacy, Al-Azhar University, Cairo 11884, Egypt

## Abstract

The title compound, C_23_H_17_FN_2_O_3_, crystallizes with two independent mol­ecules in the asymmetric unit. The mol­ecules are connected by pairs of N—H⋯O hydrogen bonds and have slightly different conformations, as indicated by the dihedral angles between the central imidazolidine-2,4-dione ring and its three substituents. In one mol­ecule, these are 60.56 (1) and 82.66 (9)° to the phenyl rings and 84.35 (16)° to the 2-(4-fluoro­phen­yl)-2-oxoethyl side chain. In the other mol­ecule, the corresponding angles are 66.35 (10), 84.94 (9) and 86.31 (16)°. In the crystal, weak C—H⋯O inter­actions leading to a three-dimensional supramolecular architecture.

## Related literature   

For studies on the biological applications of hydantoin deriv­atives, see: El-Deeb *et al.* (2010 ▶); Rajic *et al.* (2006 ▶); Carmi *et al.* (2006 ▶); Sergent *et al.* (2008 ▶). For related crystal structures, see: Delgado *et al.* (2007 ▶); Roszak & Weaver (1998 ▶); Kashif *et al.* (2008 ▶); Coquerel *et al.* (1993 ▶); SethuSankar *et al.* (2002 ▶); Eknoian *et al.* (1999 ▶); Ciechanowicz-Rutkowska *et al.* (1994 ▶).
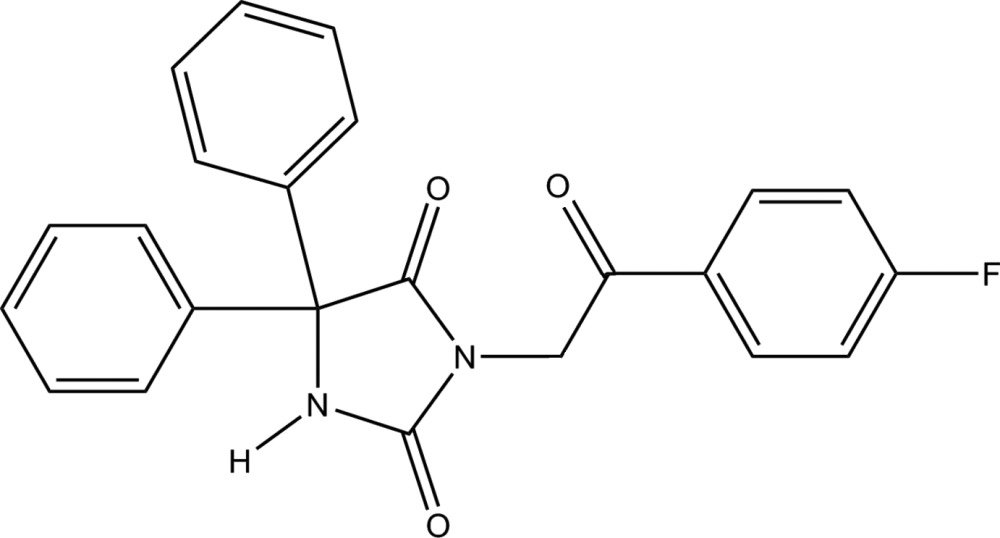


## Experimental   

### 

#### Crystal data  

C_23_H_17_FN_2_O_3_
*M* *_r_* = 388.39
Orthorhombic, 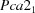

*a* = 19.9622 (16) Å
*b* = 8.0484 (6) Å
*c* = 22.8969 (18) Å
*V* = 3678.7 (5) Å^3^
*Z* = 8
Mo *K*α radiation
μ = 0.10 mm^−1^
*T* = 100 K
0.28 × 0.06 × 0.05 mm

#### Data collection  

Bruker SMART APEXII CCD diffractometer
Absorption correction: multi-scan (*SADABS*; Sheldrick, 2009 ▶) *T*_min_ = 0.778, *T*_max_ = 0.995
29816 measured reflections
8117 independent reflections
6536 reflections with *I* > 2σ(*I*)
*R*_int_ = 0.061

#### Refinement  

*R*[*F* ^2^ > 2σ(*F* ^2^)] = 0.041
*wR*(*F* ^2^) = 0.084
*S* = 1.04
8117 reflections
523 parameters
1 restraint
H-atom parameters constrained
Δρ_max_ = 0.20 e Å^−3^
Δρ_min_ = −0.18 e Å^−3^

### 

Data collection: *APEX2* (Bruker, 2010 ▶); cell refinement: *SAINT* (Bruker, 2009 ▶); data reduction: *SAINT*; program(s) used to solve structure: *SHELXS97* (Sheldrick, 2008 ▶); program(s) used to refine structure: *SHELXL97* (Sheldrick, 2008 ▶); molecular graphics: *SHELXTL* (Sheldrick, 2008 ▶); software used to prepare material for publication: *SHELXTL*.

## Supplementary Material

Crystal structure: contains datablock(s) I, global. DOI: 10.1107/S1600536814001743/fj2658sup1.cif

Structure factors: contains datablock(s) I. DOI: 10.1107/S1600536814001743/fj2658Isup2.hkl

Supporting information file. DOI: 10.1107/S1600536814001743/fj2658Isup3.cml

CCDC reference:

Additional supporting information:  crystallographic information; 3D view; checkCIF report

## Figures and Tables

**Table 1:** Hydrogen-bond geometry (Å, °)

*D*—H⋯*A*	*D*—H	H⋯*A*	*D*⋯*A*	*D*—H⋯*A*
N2—H2⋯O4	0.98	1.93	2.900 (3)	171
C16—H16*A*⋯O4^i^	0.99	2.52	3.385 (4)	146
C20—H20⋯O6^ii^	0.95	2.62	3.275 (4)	126
N4—H4⋯O1	0.95	1.98	2.931 (3)	174
C39—H39*B*⋯O1^iii^	0.99	2.57	3.403 (4)	141

Symmetry codes: (i) 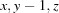; (ii) 
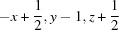
; (iii) 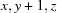.

**Fig. 1.**
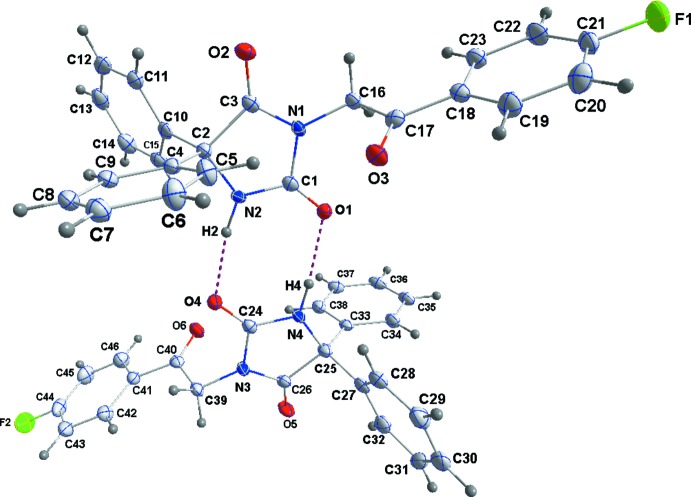
Perspective view of the asymmetric unit showing the intermolecular N—H···O hydrogen bonds as dotted lines. Displacement ellipsoids are drawn at the 50% probability level.

**Fig. 2.**
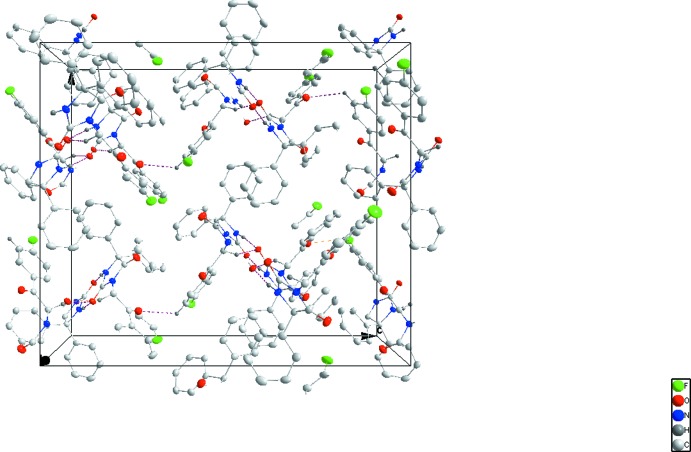
Packing diagram viewed along b with intermolecular N—H···O and C—H···O interactions shown as dotted lines.

## supplementary crystallographic information

### 1. Comment

Hydantoins comprise an important class of compounds which have long attracted attention owing to their remarkable biological and pharmacological properties including antitumor and antiviral activities, insulinotropic properties and EGFR inhibitors (El-Deeb *et al.*, 2010; Rajic *et al.*, 2006; Carmi *et al.*, 2006; Sergent *et al.*, 2008). As a continuation of our studies on hydantoin derivatives we report the successful synthesis of the title compound, 3-(2-(4-fluorophenyl)-2-oxoethyl)-5,5-diphenylimidazolidine-2,4-dione, by the reaction of 5,5-diphenylhydantoin and 4-fluorophenacyl chloride. The compound crystallizes with two independent molecules (A and B) in the asymmetric unit which are connected by pairwise N—H···O hydrogen bonds (Table 1). In addition there are weak C—H···O intermolecular interactions. The two molecules have somewhat different conformations as indicated by the dihedral angles between the central imidazolidine-2,4-dione ring and its three substituents. In molecule A these are 60.56 (1) and 82.66 (9)° to the phenyl rings based on C10 and C4, respectively and 84.35 (16)° to the 2-(4-fluorophenyl)-2-oxoethyl side chain. In molecule B, the corresponding angles are 66.35 (10), 84.94 (9) and 86.31 (16)°.

### 2. Experimental

A mixture of 5,5-diphenylhydantoin (1 mmol) and K_2_CO_3_ (1.1 mmol) was stirred in acetone (20 ml) at room temperature for 20 min. To the resulting mixture 4-fluorophenacyl chloride (1.0 mmol) in acetone (5 ml) was added dropwise over a period of 10 min. The reaction mixture was further stirred at room temperature for 6 h. The separated solid was then filtered, washed with cold water, dried and crystallized from MeOH/CHCl_3_. M. P. 251–252°C. Yield: 99%. ^1^H NMR (DMSO-d_6_): δ 9.78 (s, 1H, NH), 8.18–8.15 (q, 2H, J = 7.5 Hz, Ar—H), 7.47–7.39 (m, 12H, Ar—H), 5.08 (s, 2H, –CH_2_). ^13^C NMR (DMSO-d_6_): δ 190.89, 173.36, 166.57, 164.56, 154.84, 139.50, 131.38, 131.30, 130.72, 128.52, 128.23, 126.86, 126.54, 116.17, 116.00, 62.91, 44.77

### Crystal data

**Table:**

C_23_H_17_FN_2_O_3_	*F*(000) = 1616
*M**_r_* = 388.39	*D*_x_ = 1.403 Mg m^−^^3^
Orthorhombic, *P**c**a*2_1_	Mo *K*α radiation, λ = 0.71073 Å
Hall symbol: P 2c -2ac	Cell parameters from 9958 reflections
*a* = 19.9622 (16) Å	θ = 2.2–26.5°
*b* = 8.0484 (6) Å	µ = 0.10 mm^−^^1^
*c* = 22.8969 (18) Å	*T* = 100 K
*V* = 3678.7 (5) Å^3^	Column, colourless
*Z* = 8	0.28 × 0.06 × 0.05 mm

### Data collection

**Table:**

Bruker SMART APEXII CCD diffractometer	8117 independent reflections
Radiation source: fine-focus sealed tube	6536 reflections with *I* > 2σ(*I*)
Graphite monochromator	*R*_int_ = 0.061
Detector resolution: 8.3660 pixels mm^-1^	θ_max_ = 27.2°, θ_min_ = 1.8°
φ and ω scans	*h* = −25→25
Absorption correction: multi-scan (*SADABS*; Sheldrick, 2009)	*k* = −10→10
*T*_min_ = 0.778, *T*_max_ = 0.995	*l* = −29→29
29816 measured reflections	

### Refinement

**Table:**

Refinement on *F*^2^	Primary atom site location: structure-invariant direct methods
Least-squares matrix: full	Secondary atom site location: difference Fourier map
*R*[*F*^2^ > 2σ(*F*^2^)] = 0.041	Hydrogen site location: mixed
*wR*(*F*^2^) = 0.084	H-atom parameters constrained
*S* = 1.04	*w* = 1/[σ^2^(*F*_o_^2^) + (0.0262*P*)^2^ + 0.2586*P*] where *P* = (*F*_o_^2^ + 2*F*_c_^2^)/3
8117 reflections	(Δ/σ)_max_ < 0.001
523 parameters	Δρ_max_ = 0.20 e Å^−^^3^
1 restraint	Δρ_min_ = −0.18 e Å^−^^3^

### Special details

**Table:**

Experimental. The diffraction data were collected in three sets of 606 frames (0.3° width in ω) at φ = 0, 120 and 240°. A scan time of 60 sec/frame was used.
Geometry. All e.s.d.'s (except the e.s.d. in the dihedral angle between two l.s. planes) are estimated using the full covariance matrix. The cell e.s.d.'s are taken into account individually in the estimation of e.s.d.'s in distances, angles and torsion angles; correlations between e.s.d.'s in cell parameters are only used when they are defined by crystal symmetry. An approximate (isotropic) treatment of cell e.s.d.'s is used for estimating e.s.d.'s involving l.s. planes.
Refinement. Refinement of *F*^2^ against ALL reflections. The weighted *R*-factor *wR* and goodness of fit *S* are based on *F*^2^, conventional *R*-factors *R* are based on *F*, with *F* set to zero for negative *F*^2^. The threshold expression of *F*^2^ > σ(*F*^2^) is used only for calculating *R*-factors(gt) *etc*. and is not relevant to the choice of reflections for refinement. *R*-factors based on *F*^2^ are statistically about twice as large as those based on *F*, and *R*- factors based on ALL data will be even larger. H-atoms attached to carbon were placed in calculated positions (C—H = 0.95 - 0.98 Å) while those attached to nitrogen were placed in locations derived from a difference map. All were included as riding contributions with isotropic displacement parameters 1.2 - 1.5 times those of the attached atoms. Friedel opposites were merged in the final refinement.

### Fractional atomic coordinates and isotropic or equivalent isotropic displacement parameters (Å^2^)

**Table:**

	*x*	*y*	*z*	*U*_iso_*/*U*_eq_	
F1	0.36506 (10)	−0.8027 (2)	0.60459 (9)	0.0357 (5)	
O1	0.19858 (10)	−0.0057 (2)	0.42585 (9)	0.0190 (5)	
O2	0.03918 (11)	−0.1218 (3)	0.56297 (10)	0.0259 (5)	
O3	0.22739 (11)	−0.1078 (2)	0.56211 (9)	0.0225 (5)	
N1	0.12052 (12)	−0.1090 (3)	0.49185 (11)	0.0164 (5)	
N2	0.11049 (12)	0.1514 (3)	0.46195 (11)	0.0164 (5)	
H2	0.1223	0.2522	0.4402	0.020*	
C1	0.14846 (15)	0.0142 (4)	0.45623 (13)	0.0163 (6)	
C2	0.05492 (15)	0.1311 (3)	0.50328 (13)	0.0166 (6)	
C3	0.06822 (15)	−0.0484 (4)	0.52471 (13)	0.0172 (7)	
C4	0.06107 (15)	0.2530 (4)	0.55430 (13)	0.0170 (6)	
C5	0.11150 (16)	0.2311 (4)	0.59527 (14)	0.0238 (7)	
H5	0.1404	0.1377	0.5924	0.029*	
C6	0.12004 (16)	0.3443 (4)	0.64032 (14)	0.0258 (8)	
H6	0.1546	0.3278	0.6683	0.031*	
C7	0.07806 (16)	0.4822 (4)	0.64465 (14)	0.0222 (7)	
H7	0.0841	0.5603	0.6753	0.027*	
C8	0.02776 (16)	0.5045 (4)	0.60409 (14)	0.0215 (7)	
H8	−0.0011	0.5979	0.6071	0.026*	
C9	0.01895 (15)	0.3910 (3)	0.55873 (14)	0.0187 (7)	
H9	−0.0157	0.4076	0.5309	0.022*	
C10	−0.01380 (15)	0.1376 (3)	0.47312 (14)	0.0181 (6)	
C11	−0.07052 (15)	0.0907 (4)	0.50442 (15)	0.0218 (7)	
H11	−0.0662	0.0525	0.5435	0.026*	
C12	−0.13317 (16)	0.0997 (4)	0.47873 (16)	0.0279 (8)	
H12	−0.1716	0.0647	0.4999	0.033*	
C13	−0.14007 (16)	0.1593 (4)	0.42243 (16)	0.0296 (8)	
H13	−0.1833	0.1674	0.4053	0.036*	
C14	−0.08425 (17)	0.2069 (4)	0.39121 (16)	0.0273 (8)	
H14	−0.0890	0.2481	0.3525	0.033*	
C15	−0.02101 (16)	0.1946 (4)	0.41633 (14)	0.0223 (7)	
H15	0.0175	0.2255	0.3945	0.027*	
C16	0.15318 (14)	−0.2671 (3)	0.50337 (14)	0.0171 (6)	
H16A	0.1679	−0.3171	0.4660	0.020*	
H16B	0.1208	−0.3440	0.5219	0.020*	
C17	0.21364 (15)	−0.2450 (4)	0.54340 (13)	0.0172 (6)	
C18	0.25310 (15)	−0.3954 (4)	0.55861 (14)	0.0183 (6)	
C19	0.30493 (16)	−0.3805 (4)	0.59887 (15)	0.0245 (7)	
H19	0.3145	−0.2751	0.6156	0.029*	
C20	0.34283 (17)	−0.5176 (4)	0.61489 (15)	0.0280 (8)	
H20	0.3782	−0.5086	0.6425	0.034*	
C21	0.32710 (16)	−0.6676 (4)	0.58915 (14)	0.0235 (7)	
C22	0.27679 (16)	−0.6890 (4)	0.54978 (14)	0.0248 (7)	
H22	0.2677	−0.7950	0.5333	0.030*	
C23	0.23913 (16)	−0.5508 (4)	0.53446 (14)	0.0221 (7)	
H23	0.2035	−0.5620	0.5073	0.027*	
F2	0.00915 (10)	1.2515 (3)	0.20897 (10)	0.0411 (6)	
O4	0.15962 (10)	0.4378 (2)	0.39817 (9)	0.0194 (5)	
O5	0.31701 (11)	0.5522 (3)	0.25991 (10)	0.0250 (5)	
O6	0.13476 (10)	0.5430 (3)	0.25498 (10)	0.0251 (5)	
N3	0.23672 (12)	0.5419 (3)	0.33196 (11)	0.0175 (6)	
N4	0.25317 (12)	0.2895 (3)	0.36792 (11)	0.0170 (5)	
H4	0.2385	0.1925	0.3880	0.020*	
C24	0.21119 (15)	0.4210 (3)	0.36944 (13)	0.0153 (6)	
C25	0.30877 (14)	0.3122 (4)	0.32701 (13)	0.0180 (6)	
C26	0.29022 (15)	0.4827 (4)	0.30006 (13)	0.0167 (6)	
C27	0.37645 (15)	0.3334 (3)	0.35772 (14)	0.0200 (7)	
C28	0.38363 (16)	0.3039 (4)	0.41717 (15)	0.0227 (7)	
H28	0.3460	0.2709	0.4397	0.027*	
C29	0.44577 (17)	0.3226 (4)	0.44383 (16)	0.0297 (8)	
H29	0.4506	0.2995	0.4843	0.036*	
C30	0.50060 (17)	0.3748 (4)	0.41166 (17)	0.0307 (8)	
H30	0.5427	0.3904	0.4301	0.037*	
C31	0.49369 (16)	0.4041 (4)	0.35262 (16)	0.0259 (8)	
H31	0.5314	0.4392	0.3305	0.031*	
C32	0.43241 (16)	0.3827 (4)	0.32518 (15)	0.0231 (7)	
H32	0.4284	0.4015	0.2844	0.028*	
C33	0.30875 (15)	0.1754 (4)	0.28071 (13)	0.0168 (6)	
C34	0.35807 (16)	0.0530 (4)	0.27972 (14)	0.0211 (7)	
H34	0.3937	0.0567	0.3072	0.025*	
C35	0.35537 (16)	−0.0742 (4)	0.23886 (14)	0.0234 (7)	
H35	0.3890	−0.1576	0.2386	0.028*	
C36	0.30384 (17)	−0.0796 (4)	0.19862 (14)	0.0241 (7)	
H36	0.3021	−0.1672	0.1709	0.029*	
C37	0.25469 (17)	0.0418 (4)	0.19852 (14)	0.0238 (7)	
H37	0.2196	0.0385	0.1705	0.029*	
C38	0.25707 (16)	0.1687 (4)	0.23982 (14)	0.0213 (7)	
H38	0.2232	0.2515	0.2401	0.026*	
C39	0.20369 (15)	0.6984 (4)	0.31980 (14)	0.0183 (7)	
H39A	0.2369	0.7783	0.3043	0.022*	
H39B	0.1855	0.7445	0.3566	0.022*	
C40	0.14699 (15)	0.6786 (4)	0.27591 (13)	0.0176 (7)	
C41	0.10893 (15)	0.8310 (4)	0.26019 (14)	0.0183 (6)	
C42	0.11861 (16)	0.9804 (4)	0.28937 (14)	0.0216 (7)	
H42	0.1490	0.9853	0.3212	0.026*	
C43	0.08436 (16)	1.1226 (4)	0.27246 (15)	0.0248 (7)	
H43	0.0907	1.2249	0.2924	0.030*	
C44	0.04113 (16)	1.1111 (4)	0.22618 (15)	0.0273 (8)	
C45	0.02944 (18)	0.9670 (4)	0.19629 (15)	0.0311 (8)	
H45	−0.0010	0.9643	0.1644	0.037*	
C46	0.06323 (17)	0.8249 (4)	0.21373 (15)	0.0276 (8)	
H46	0.0554	0.7228	0.1941	0.033*	

### Atomic displacement parameters (Å^2^)

**Table:**

	*U*^11^	*U*^22^	*U*^33^	*U*^12^	*U*^13^	*U*^23^
F1	0.0353 (11)	0.0278 (11)	0.0441 (13)	0.0120 (9)	−0.0032 (10)	0.0097 (10)
O1	0.0194 (11)	0.0165 (11)	0.0212 (11)	0.0015 (8)	0.0052 (10)	−0.0014 (9)
O2	0.0307 (13)	0.0168 (11)	0.0302 (13)	−0.0003 (9)	0.0101 (11)	0.0056 (10)
O3	0.0282 (13)	0.0146 (11)	0.0247 (12)	0.0001 (9)	−0.0007 (10)	−0.0042 (9)
N1	0.0198 (13)	0.0098 (12)	0.0196 (14)	0.0023 (10)	0.0026 (11)	−0.0005 (10)
N2	0.0195 (13)	0.0110 (12)	0.0188 (14)	−0.0007 (10)	0.0056 (11)	0.0014 (10)
C1	0.0191 (16)	0.0137 (15)	0.0162 (15)	−0.0013 (12)	−0.0006 (13)	−0.0021 (12)
C2	0.0184 (15)	0.0128 (15)	0.0185 (15)	0.0007 (12)	0.0042 (13)	0.0011 (13)
C3	0.0197 (16)	0.0134 (15)	0.0187 (16)	−0.0012 (12)	−0.0013 (13)	0.0001 (13)
C4	0.0206 (16)	0.0126 (14)	0.0179 (15)	−0.0008 (12)	0.0055 (13)	0.0004 (12)
C5	0.0273 (18)	0.0199 (17)	0.0243 (17)	0.0067 (14)	−0.0023 (15)	0.0004 (14)
C6	0.0292 (19)	0.0265 (19)	0.0218 (18)	0.0067 (14)	−0.0029 (15)	−0.0027 (14)
C7	0.0276 (17)	0.0174 (16)	0.0217 (17)	−0.0013 (13)	0.0028 (14)	−0.0028 (13)
C8	0.0233 (17)	0.0143 (15)	0.0267 (17)	0.0004 (12)	0.0059 (15)	0.0007 (13)
C9	0.0207 (17)	0.0142 (15)	0.0211 (16)	−0.0006 (12)	0.0019 (14)	0.0026 (13)
C10	0.0197 (16)	0.0098 (14)	0.0250 (17)	0.0011 (12)	0.0000 (14)	−0.0057 (13)
C11	0.0238 (18)	0.0153 (16)	0.0264 (18)	0.0007 (13)	0.0050 (14)	−0.0019 (13)
C12	0.0216 (18)	0.0184 (17)	0.044 (2)	−0.0017 (13)	0.0065 (16)	−0.0054 (16)
C13	0.0221 (18)	0.0186 (17)	0.048 (2)	0.0006 (13)	−0.0091 (17)	−0.0054 (16)
C14	0.0314 (19)	0.0208 (18)	0.0298 (19)	0.0001 (14)	−0.0111 (16)	0.0000 (15)
C15	0.0254 (17)	0.0145 (15)	0.0269 (18)	−0.0015 (13)	−0.0008 (15)	−0.0029 (13)
C16	0.0192 (15)	0.0119 (15)	0.0201 (16)	0.0004 (12)	0.0020 (13)	0.0002 (12)
C17	0.0188 (16)	0.0172 (15)	0.0155 (15)	−0.0002 (12)	0.0029 (12)	−0.0016 (12)
C18	0.0215 (16)	0.0168 (15)	0.0167 (15)	−0.0006 (12)	0.0020 (13)	0.0016 (12)
C19	0.0260 (18)	0.0193 (17)	0.0281 (18)	0.0014 (13)	−0.0026 (15)	−0.0029 (14)
C20	0.0262 (19)	0.033 (2)	0.0250 (18)	0.0032 (15)	−0.0048 (15)	−0.0013 (15)
C21	0.0254 (18)	0.0201 (17)	0.0249 (18)	0.0091 (13)	0.0029 (14)	0.0063 (14)
C22	0.0309 (19)	0.0139 (16)	0.0294 (18)	0.0018 (13)	−0.0009 (16)	−0.0013 (14)
C23	0.0249 (17)	0.0179 (16)	0.0236 (17)	−0.0013 (13)	−0.0013 (14)	0.0013 (13)
F2	0.0370 (12)	0.0305 (11)	0.0559 (14)	0.0110 (10)	−0.0066 (10)	0.0189 (10)
O4	0.0199 (11)	0.0165 (11)	0.0219 (12)	0.0017 (9)	0.0046 (9)	0.0008 (9)
O5	0.0319 (12)	0.0170 (11)	0.0260 (12)	0.0001 (9)	0.0091 (11)	0.0056 (10)
O6	0.0298 (12)	0.0166 (11)	0.0291 (13)	0.0006 (9)	−0.0014 (11)	−0.0060 (10)
N3	0.0185 (13)	0.0133 (13)	0.0208 (14)	0.0026 (10)	0.0037 (11)	0.0024 (10)
N4	0.0194 (13)	0.0116 (12)	0.0198 (13)	0.0026 (10)	0.0054 (11)	0.0037 (10)
C24	0.0180 (16)	0.0135 (15)	0.0142 (14)	−0.0001 (12)	−0.0028 (13)	−0.0016 (12)
C25	0.0194 (16)	0.0145 (15)	0.0201 (16)	−0.0004 (12)	0.0044 (13)	0.0013 (13)
C26	0.0197 (16)	0.0123 (15)	0.0180 (15)	−0.0015 (12)	0.0017 (13)	−0.0012 (12)
C27	0.0228 (16)	0.0107 (14)	0.0265 (18)	0.0011 (12)	0.0003 (14)	−0.0040 (13)
C28	0.0255 (17)	0.0143 (15)	0.0284 (19)	0.0003 (13)	−0.0002 (15)	−0.0019 (13)
C29	0.036 (2)	0.0209 (18)	0.033 (2)	0.0017 (15)	−0.0112 (16)	0.0033 (14)
C30	0.0240 (18)	0.0220 (18)	0.046 (2)	0.0022 (14)	−0.0094 (17)	−0.0061 (16)
C31	0.0183 (17)	0.0204 (17)	0.039 (2)	−0.0012 (13)	0.0044 (15)	−0.0076 (15)
C32	0.0262 (17)	0.0169 (16)	0.0262 (18)	0.0028 (13)	0.0034 (15)	−0.0054 (14)
C33	0.0191 (16)	0.0120 (15)	0.0194 (15)	−0.0026 (12)	0.0051 (13)	0.0014 (12)
C34	0.0233 (17)	0.0148 (15)	0.0253 (17)	0.0014 (13)	0.0033 (14)	−0.0019 (13)
C35	0.0302 (18)	0.0136 (16)	0.0264 (18)	0.0004 (13)	0.0057 (15)	−0.0013 (13)
C36	0.037 (2)	0.0158 (16)	0.0192 (16)	−0.0038 (14)	0.0039 (15)	−0.0020 (13)
C37	0.0273 (18)	0.0221 (17)	0.0220 (17)	−0.0043 (14)	−0.0032 (14)	0.0002 (14)
C38	0.0224 (16)	0.0164 (15)	0.0250 (17)	0.0002 (13)	0.0015 (14)	0.0023 (13)
C39	0.0213 (16)	0.0119 (15)	0.0216 (16)	0.0020 (12)	0.0012 (13)	0.0014 (12)
C40	0.0202 (16)	0.0159 (15)	0.0167 (16)	−0.0012 (12)	0.0045 (13)	0.0029 (12)
C41	0.0174 (15)	0.0177 (16)	0.0197 (16)	0.0010 (12)	0.0009 (14)	0.0027 (13)
C42	0.0225 (17)	0.0204 (17)	0.0219 (17)	0.0005 (13)	−0.0025 (13)	0.0037 (13)
C43	0.0238 (17)	0.0172 (16)	0.034 (2)	0.0012 (13)	0.0009 (15)	0.0044 (14)
C44	0.0227 (18)	0.0266 (19)	0.033 (2)	0.0081 (14)	0.0036 (15)	0.0137 (16)
C45	0.0270 (19)	0.036 (2)	0.030 (2)	0.0045 (16)	−0.0083 (16)	0.0057 (17)
C46	0.0282 (19)	0.0264 (19)	0.0281 (19)	0.0007 (14)	−0.0044 (15)	−0.0010 (15)

### Geometric parameters (Å, º)

**Table:**

F1—C21	1.371 (3)	F2—C44	1.357 (4)
O1—C1	1.229 (3)	O4—C24	1.229 (3)
O2—C3	1.205 (3)	O5—C26	1.202 (3)
O3—C17	1.216 (3)	O6—C40	1.217 (3)
N1—C3	1.376 (4)	N3—C26	1.379 (4)
N1—C1	1.400 (4)	N3—C24	1.394 (4)
N1—C16	1.454 (3)	N3—C39	1.449 (4)
N2—C1	1.345 (4)	N4—C24	1.350 (4)
N2—C2	1.467 (4)	N4—C25	1.464 (4)
N2—H2	0.9814	N4—H4	0.9521
C2—C4	1.530 (4)	C25—C33	1.528 (4)
C2—C10	1.537 (4)	C25—C27	1.533 (4)
C2—C3	1.549 (4)	C25—C26	1.550 (4)
C4—C5	1.387 (4)	C27—C28	1.389 (4)
C4—C9	1.397 (4)	C27—C32	1.400 (4)
C5—C6	1.387 (4)	C28—C29	1.391 (5)
C5—H5	0.9500	C28—H28	0.9500
C6—C7	1.395 (4)	C29—C30	1.385 (5)
C6—H6	0.9500	C29—H29	0.9500
C7—C8	1.379 (4)	C30—C31	1.379 (5)
C7—H7	0.9500	C30—H30	0.9500
C8—C9	1.394 (4)	C31—C32	1.386 (5)
C8—H8	0.9500	C31—H31	0.9500
C9—H9	0.9500	C32—H32	0.9500
C10—C15	1.386 (4)	C33—C34	1.393 (4)
C10—C11	1.392 (4)	C33—C38	1.394 (4)
C11—C12	1.384 (4)	C34—C35	1.388 (4)
C11—H11	0.9500	C34—H34	0.9500
C12—C13	1.382 (5)	C35—C36	1.382 (5)
C12—H12	0.9500	C35—H35	0.9500
C13—C14	1.378 (5)	C36—C37	1.385 (5)
C13—H13	0.9500	C36—H36	0.9500
C14—C15	1.391 (4)	C37—C38	1.393 (4)
C14—H14	0.9500	C37—H37	0.9500
C15—H15	0.9500	C38—H38	0.9500
C16—C17	1.526 (4)	C39—C40	1.522 (4)
C16—H16A	0.9900	C39—H39A	0.9900
C16—H16B	0.9900	C39—H39B	0.9900
C17—C18	1.486 (4)	C40—C41	1.487 (4)
C18—C19	1.391 (4)	C41—C42	1.389 (4)
C18—C23	1.395 (4)	C41—C46	1.402 (4)
C19—C20	1.388 (5)	C42—C43	1.388 (4)
C19—H19	0.9500	C42—H42	0.9500
C20—C21	1.379 (5)	C43—C44	1.370 (5)
C20—H20	0.9500	C43—H43	0.9500
C21—C22	1.361 (5)	C44—C45	1.366 (5)
C22—C23	1.388 (4)	C45—C46	1.386 (5)
C22—H22	0.9500	C45—H45	0.9500
C23—H23	0.9500	C46—H46	0.9500
			
C3—N1—C1	111.7 (2)	C26—N3—C24	111.6 (2)
C3—N1—C16	123.4 (2)	C26—N3—C39	123.4 (2)
C1—N1—C16	123.2 (2)	C24—N3—C39	124.0 (2)
C1—N2—C2	113.4 (2)	C24—N4—C25	112.9 (2)
C1—N2—H2	119.6	C24—N4—H4	116.1
C2—N2—H2	126.9	C25—N4—H4	130.1
O1—C1—N2	128.4 (3)	O4—C24—N4	128.3 (3)
O1—C1—N1	124.2 (3)	O4—C24—N3	124.0 (3)
N2—C1—N1	107.5 (2)	N4—C24—N3	107.7 (2)
N2—C2—C4	111.1 (2)	N4—C25—C33	110.7 (2)
N2—C2—C10	112.4 (2)	N4—C25—C27	112.9 (2)
C4—C2—C10	113.1 (2)	C33—C25—C27	113.5 (2)
N2—C2—C3	100.3 (2)	N4—C25—C26	100.6 (2)
C4—C2—C3	110.0 (2)	C33—C25—C26	111.2 (2)
C10—C2—C3	109.1 (2)	C27—C25—C26	107.1 (2)
O2—C3—N1	126.1 (3)	O5—C26—N3	126.1 (3)
O2—C3—C2	127.2 (3)	O5—C26—C25	127.7 (3)
N1—C3—C2	106.7 (2)	N3—C26—C25	106.3 (2)
C5—C4—C9	119.3 (3)	C28—C27—C32	119.2 (3)
C5—C4—C2	119.5 (3)	C28—C27—C25	121.4 (3)
C9—C4—C2	121.1 (3)	C32—C27—C25	119.4 (3)
C6—C5—C4	120.6 (3)	C27—C28—C29	120.2 (3)
C6—C5—H5	119.7	C27—C28—H28	119.9
C4—C5—H5	119.7	C29—C28—H28	119.9
C5—C6—C7	120.1 (3)	C30—C29—C28	120.3 (3)
C5—C6—H6	119.9	C30—C29—H29	119.9
C7—C6—H6	119.9	C28—C29—H29	119.9
C8—C7—C6	119.5 (3)	C31—C30—C29	119.6 (3)
C8—C7—H7	120.2	C31—C30—H30	120.2
C6—C7—H7	120.2	C29—C30—H30	120.2
C7—C8—C9	120.6 (3)	C30—C31—C32	120.8 (3)
C7—C8—H8	119.7	C30—C31—H31	119.6
C9—C8—H8	119.7	C32—C31—H31	119.6
C8—C9—C4	119.9 (3)	C31—C32—C27	119.9 (3)
C8—C9—H9	120.0	C31—C32—H32	120.1
C4—C9—H9	120.0	C27—C32—H32	120.1
C15—C10—C11	119.2 (3)	C34—C33—C38	119.0 (3)
C15—C10—C2	121.7 (3)	C34—C33—C25	121.4 (3)
C11—C10—C2	119.0 (3)	C38—C33—C25	119.6 (3)
C12—C11—C10	120.1 (3)	C35—C34—C33	120.3 (3)
C12—C11—H11	119.9	C35—C34—H34	119.8
C10—C11—H11	119.9	C33—C34—H34	119.8
C13—C12—C11	120.3 (3)	C36—C35—C34	120.1 (3)
C13—C12—H12	119.8	C36—C35—H35	119.9
C11—C12—H12	119.8	C34—C35—H35	119.9
C14—C13—C12	120.0 (3)	C35—C36—C37	120.4 (3)
C14—C13—H13	120.0	C35—C36—H36	119.8
C12—C13—H13	120.0	C37—C36—H36	119.8
C13—C14—C15	120.0 (3)	C36—C37—C38	119.5 (3)
C13—C14—H14	120.0	C36—C37—H37	120.3
C15—C14—H14	120.0	C38—C37—H37	120.3
C10—C15—C14	120.4 (3)	C37—C38—C33	120.7 (3)
C10—C15—H15	119.8	C37—C38—H38	119.7
C14—C15—H15	119.8	C33—C38—H38	119.7
N1—C16—C17	111.2 (2)	N3—C39—C40	112.0 (2)
N1—C16—H16A	109.4	N3—C39—H39A	109.2
C17—C16—H16A	109.4	C40—C39—H39A	109.2
N1—C16—H16B	109.4	N3—C39—H39B	109.2
C17—C16—H16B	109.4	C40—C39—H39B	109.2
H16A—C16—H16B	108.0	H39A—C39—H39B	107.9
O3—C17—C18	122.5 (3)	O6—C40—C41	122.8 (3)
O3—C17—C16	119.8 (3)	O6—C40—C39	120.2 (3)
C18—C17—C16	117.7 (2)	C41—C40—C39	117.0 (3)
C19—C18—C23	119.2 (3)	C42—C41—C46	119.0 (3)
C19—C18—C17	118.7 (3)	C42—C41—C40	121.8 (3)
C23—C18—C17	122.1 (3)	C46—C41—C40	119.2 (3)
C20—C19—C18	120.8 (3)	C43—C42—C41	120.7 (3)
C20—C19—H19	119.6	C43—C42—H42	119.6
C18—C19—H19	119.6	C41—C42—H42	119.6
C21—C20—C19	117.3 (3)	C44—C43—C42	118.0 (3)
C21—C20—H20	121.3	C44—C43—H43	121.0
C19—C20—H20	121.3	C42—C43—H43	121.0
C22—C21—F1	118.6 (3)	F2—C44—C45	118.7 (3)
C22—C21—C20	124.2 (3)	F2—C44—C43	117.7 (3)
F1—C21—C20	117.2 (3)	C45—C44—C43	123.5 (3)
C21—C22—C23	117.8 (3)	C44—C45—C46	118.2 (3)
C21—C22—H22	121.1	C44—C45—H45	120.9
C23—C22—H22	121.1	C46—C45—H45	120.9
C22—C23—C18	120.7 (3)	C45—C46—C41	120.4 (3)
C22—C23—H23	119.7	C45—C46—H46	119.8
C18—C23—H23	119.7	C41—C46—H46	119.8
			
C2—N2—C1—O1	−178.8 (3)	C25—N4—C24—O4	178.3 (3)
C2—N2—C1—N1	1.5 (3)	C25—N4—C24—N3	−2.2 (3)
C3—N1—C1—O1	174.8 (3)	C26—N3—C24—O4	−172.6 (3)
C16—N1—C1—O1	9.1 (5)	C39—N3—C24—O4	−3.5 (4)
C3—N1—C1—N2	−5.5 (3)	C26—N3—C24—N4	7.9 (3)
C16—N1—C1—N2	−171.1 (2)	C39—N3—C24—N4	177.0 (3)
C1—N2—C2—C4	118.8 (3)	C24—N4—C25—C33	−121.1 (3)
C1—N2—C2—C10	−113.3 (3)	C24—N4—C25—C27	110.4 (3)
C1—N2—C2—C3	2.5 (3)	C24—N4—C25—C26	−3.4 (3)
C1—N1—C3—O2	−172.5 (3)	C24—N3—C26—O5	170.9 (3)
C16—N1—C3—O2	−6.9 (5)	C39—N3—C26—O5	1.8 (5)
C1—N1—C3—C2	6.9 (3)	C24—N3—C26—C25	−9.9 (3)
C16—N1—C3—C2	172.5 (2)	C39—N3—C26—C25	−179.0 (2)
N2—C2—C3—O2	174.0 (3)	N4—C25—C26—O5	−173.0 (3)
C4—C2—C3—O2	56.8 (4)	C33—C25—C26—O5	−55.7 (4)
C10—C2—C3—O2	−67.8 (4)	C27—C25—C26—O5	68.8 (4)
N2—C2—C3—N1	−5.5 (3)	N4—C25—C26—N3	7.8 (3)
C4—C2—C3—N1	−122.6 (3)	C33—C25—C26—N3	125.1 (3)
C10—C2—C3—N1	112.7 (3)	C27—C25—C26—N3	−110.3 (3)
N2—C2—C4—C5	−70.1 (3)	N4—C25—C27—C28	10.6 (4)
C10—C2—C4—C5	162.3 (3)	C33—C25—C27—C28	−116.5 (3)
C3—C2—C4—C5	40.0 (4)	C26—C25—C27—C28	120.4 (3)
N2—C2—C4—C9	106.5 (3)	N4—C25—C27—C32	−169.8 (3)
C10—C2—C4—C9	−21.0 (4)	C33—C25—C27—C32	63.2 (3)
C3—C2—C4—C9	−143.3 (3)	C26—C25—C27—C32	−60.0 (3)
C9—C4—C5—C6	0.2 (5)	C32—C27—C28—C29	−0.3 (4)
C2—C4—C5—C6	176.9 (3)	C25—C27—C28—C29	179.4 (3)
C4—C5—C6—C7	−0.4 (5)	C27—C28—C29—C30	1.6 (5)
C5—C6—C7—C8	0.5 (5)	C28—C29—C30—C31	−1.7 (5)
C6—C7—C8—C9	−0.5 (5)	C29—C30—C31—C32	0.4 (5)
C7—C8—C9—C4	0.3 (5)	C30—C31—C32—C27	1.0 (5)
C5—C4—C9—C8	−0.2 (5)	C28—C27—C32—C31	−1.0 (4)
C2—C4—C9—C8	−176.9 (3)	C25—C27—C32—C31	179.3 (3)
N2—C2—C10—C15	−13.9 (4)	N4—C25—C33—C34	−110.1 (3)
C4—C2—C10—C15	113.0 (3)	C27—C25—C33—C34	18.1 (4)
C3—C2—C10—C15	−124.2 (3)	C26—C25—C33—C34	139.0 (3)
N2—C2—C10—C11	168.5 (2)	N4—C25—C33—C38	67.5 (3)
C4—C2—C10—C11	−64.6 (3)	C27—C25—C33—C38	−164.3 (3)
C3—C2—C10—C11	58.2 (3)	C26—C25—C33—C38	−43.5 (4)
C15—C10—C11—C12	0.6 (4)	C38—C33—C34—C35	−0.5 (4)
C2—C10—C11—C12	178.3 (3)	C25—C33—C34—C35	177.1 (3)
C10—C11—C12—C13	−1.7 (5)	C33—C34—C35—C36	0.4 (5)
C11—C12—C13—C14	1.3 (5)	C34—C35—C36—C37	0.3 (5)
C12—C13—C14—C15	0.1 (5)	C35—C36—C37—C38	−0.8 (5)
C11—C10—C15—C14	0.8 (4)	C36—C37—C38—C33	0.7 (5)
C2—C10—C15—C14	−176.7 (3)	C34—C33—C38—C37	−0.1 (4)
C13—C14—C15—C10	−1.2 (5)	C25—C33—C38—C37	−177.7 (3)
C3—N1—C16—C17	−92.3 (3)	C26—N3—C39—C40	88.8 (3)
C1—N1—C16—C17	71.7 (3)	C24—N3—C39—C40	−79.0 (3)
N1—C16—C17—O3	1.8 (4)	N3—C39—C40—O6	−0.9 (4)
N1—C16—C17—C18	−179.1 (2)	N3—C39—C40—C41	179.6 (2)
O3—C17—C18—C19	4.5 (5)	O6—C40—C41—C42	172.6 (3)
C16—C17—C18—C19	−174.7 (3)	C39—C40—C41—C42	−8.0 (4)
O3—C17—C18—C23	−176.5 (3)	O6—C40—C41—C46	−9.0 (4)
C16—C17—C18—C23	4.4 (4)	C39—C40—C41—C46	170.4 (3)
C23—C18—C19—C20	0.3 (5)	C46—C41—C42—C43	−0.9 (5)
C17—C18—C19—C20	179.4 (3)	C40—C41—C42—C43	177.5 (3)
C18—C19—C20—C21	0.3 (5)	C41—C42—C43—C44	−0.3 (5)
C19—C20—C21—C22	−0.6 (5)	C42—C43—C44—F2	−178.3 (3)
C19—C20—C21—F1	179.4 (3)	C42—C43—C44—C45	0.8 (5)
F1—C21—C22—C23	−179.7 (3)	F2—C44—C45—C46	179.0 (3)
C20—C21—C22—C23	0.3 (5)	C43—C44—C45—C46	−0.1 (5)
C21—C22—C23—C18	0.4 (5)	C44—C45—C46—C41	−1.1 (5)
C19—C18—C23—C22	−0.7 (5)	C42—C41—C46—C45	1.6 (5)
C17—C18—C23—C22	−179.7 (3)	C40—C41—C46—C45	−176.9 (3)

### Hydrogen-bond geometry (Å, º)

**Table:**

*D*—H···*A*	*D*—H	H···*A*	*D*···*A*	*D*—H···*A*
N2—H2···O4	0.98	1.93	2.900 (3)	171
C16—H16*A*···O4^i^	0.99	2.52	3.385 (4)	146
C20—H20···O6^ii^	0.95	2.62	3.275 (4)	126
N4—H4···O1	0.95	1.98	2.931 (3)	174
C39—H39*B*···O1^iii^	0.99	2.57	3.403 (4)	141

Symmetry codes: (i) *x*, *y*−1, *z*; (ii) −*x*+1/2, *y*−1, *z*+1/2; (iii) *x*, *y*+1, *z*.
